# Causal Associations of Modifiable Risk Factors With Migraine: Evidence From Mendelian Randomization Analysis

**DOI:** 10.7759/cureus.53448

**Published:** 2024-02-02

**Authors:** Mohammad A Jareebi, Donald M Lyall, Nawaf F Gharawi, Mohammed O Shami, Najwa Dahas, Rashed F Alfaifi, Alalaa Hakami, Mohammad A Darraj, Faris A Hakami, Mohammed H Hakami, Hassan M Almalki, Zaher T Hakami, Abdulrahman Alessa, Abdullah A Alhazmi

**Affiliations:** 1 Community and Family Medicine, Jazan University, Jazan, SAU; 2 School of Health and Wellbeing, University of Glasgow, Glasgow, GBR; 3 Faculty of Medicine, Jazan University, Jazan, SAU; 4 Medicine and Surgery, Jazan University, Jazan, SAU; 5 Directorate General of Health Affairs, Ministry of Health, Jazan, SAU; 6 Anesthesiology, Jazan University, Jazan, SAU; 7 Internal Medicine, Jazan University, Jazan, SAU

**Keywords:** headache, genetic risk factors, migraine, cheese, salad, coffee, bmi, smoking, mendelian randomization

## Abstract

Background and objectives

The exact etiology of migraine is unknown; however, it is likely a mixture of genetic and non-genetic factors including lifestyle variables like smoking and diet. This study aims to assess the causal effect of modifiable risk factors on the risk of migraine using two-sample Mendelian randomization.

Materials and methods

The study used publicly available genome-wide significant single nucleotide polymorphisms (SNPs). The study evaluated a diverse smoking exposure, encompassing age at smoking initiation, smoking intensity, and maternal smoking, alongside other pertinent risk factors, namely key dietary aspects, coffee consumption, BMI, and physical activity. Self-reported migraine was the outcome of the study. The genetic data for migraine were obtained from the FinnGen (Finland) and the UK Biobank (United Kingdom) cohorts.

Results

With sample sizes ranging from 64,949 to 632,802 for each risk factor collected from several consorts, the study included a total of 282 SNPs for all risk factors. The findings demonstrated that in the FinnGen consortium, genetically estimated dietary factors as well as BMI, were significantly associated with the risk of migraine (OR 0.765 per single unit of BMI, p = 0.011; OR 0.468 per one SD higher cheese intake, p = 0.012; OR 0.286 per one SD higher salad intake, p = 0.004, and 0.625 per one SD higher coffee consumption, p = 0.003, respectively). The results also showed that in the UK Biobank specifically, a genetically estimated history of maternal smoking was significantly associated with an elevated risk of migraine (OR=1.02, p=0.004).

Conclusions

The latest study implies a connection between maternal smoking and a heightened risk of migraines, whereas cheese intake, salad intake, coffee consumption, BMI, and physical activity are associated with a lower risk of migraine development.

## Introduction

Migraine is a debilitating neurological illness that affects around one in every seven persons globally [1]. The exact etiology of migraine is unknown; however, it is thought to be a mix of hereditary and environmental factors [2]. Smoking, certain eating habits, and obesity have all been identified as potential modifiable risk factors for migraine [3]. However, the causal relationship between these risk variables and migraine is sometimes ambiguous and confounding, particularly as cross-sectional data might bias observational research. Mendelian randomization (MR) is a statistical technique that uses genetic variations as instrumental variables to determine the causal effect of an exposure on an outcome [4,5].

Numerous research studies [6-8] have employed MR to investigate the association between smoking and migraine. These studies revealed that smoking is associated with an increased risk of migraine; however, the results varied. Smoking was connected to a higher risk of migraine in the UK Biobank (UKB) group in the United Kingdom [9]. MR investigation leveraging genetic variations related to maternal smoking as instrumental variables while controlling for a variety of potential confounders: sex, age, and BMI. In the UKB group, maternal smoking was linked to an elevated risk for migraine [10]. In terms of dietary factors, an inverse association between genetic variants associated with cheese consumption and migraine risk was found by Liu et al. in a recent MR study [11]. The mechanism by which cheese may prevent migraines is not entirely understood, however, it has been hypothesized that several cheese ingredients, such as calcium and vitamin D, may aid in the prevention of migraines [12]. More fundamentally, this suggests that dietary factors may influence migraine risk [12].

Previous studies have investigated specific risk factors in specific cohorts, whereas this study will investigate a wide variety of modifiable factors in multiple cohorts. This has advantages because UKB in isolation (as with many cohorts) has well-known biases, e.g., towards healthier and less deprived participants, which may confound associations potentially. In this study, MR analysis will be applied to examine the causal relationship between various modifiable risk factors including smoking, BMI/obesity, dietary and physical activity variables, versus the risk of migraine in two large population cohorts: FinnGen in Finland and UKB.

## Materials and methods

UKB is a relatively large prospective cohort of approximately 502,000 participants assessed in one of 22 assessment centers across England, Scotland, and Wales from 2006 to 2010. Participants underwent medical, psychological, and anthropometric phenotyping including self-reporting conditions like migraine [13]. FinnGen is a Finnish genomics research initiative that intends to collect genetic data from 500,000 Finns to examine the relationships between genes and illnesses. FinnGen was initiated in 2017 and is scheduled to be finished in 2025. Over 200,000 Finns have already provided genetic data for the study [14]. This report uses publicly available summary-level data from these cohorts. 

The present study employed a comprehensive investigation of diverse smoking exposures, encompassing age at smoking initiation, smoking intensity, and historic maternal smoking, alongside other pertinent risk factors, namely cheese intake, salad intake, coffee intake, BMI, and self-reported physical activity.

**Figure 1:**
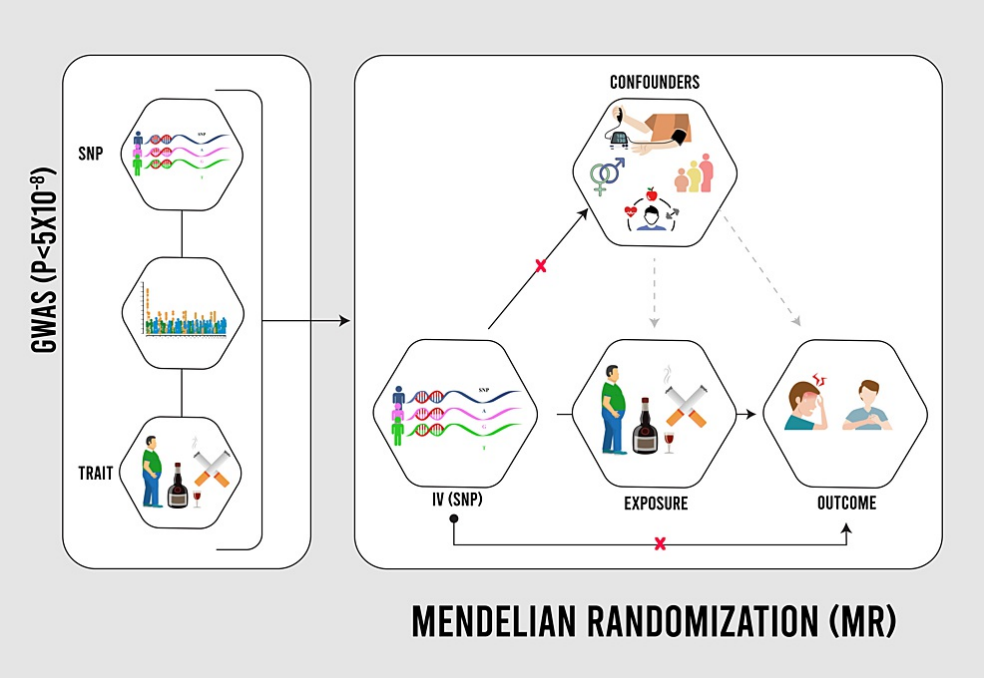
Mendelian randomization design GWAS: Genome-Wide Association Studies; SNP: single nucleotide polymorphism Image credit: Mohammad A. Jareebi (Author)

To identify relevant variables, we drew upon genome-wide significant single nucleotide polymorphisms (SNPs) previously elucidated by well-established consorts, including the UKB [15], Genome-wide Association Studies (GWAS) and Sequencing Consortium of Alcohol and Nicotine (GSCAN) use [16], Genetic Investigation of Anthropometric Traits (GIANT) [17], and Klimentidis et al.'s study [18]. SNPs represent genetic variations associated with specific traits, as ascertained through GWAS at a significance threshold of P <5x10^-8^ [19,20]. The study employed a distinct array of SNPs for each exposure, comprising 93 SNPs for smoking initiation [21], six SNPs for smoking intensity [22], seven SNPs for maternal smoking [23], 65 SNPs for cheese intake [24], 22 SNPs for salad intake [25], three SNPs for coffee intake [26], 79 SNPs for BMI [17], and seven SNPs for physical activity (Figure 1) [27].

The principal objective of this investigation was to assess migraine as a binary outcome. Genetic data [28,29] about migraine were acquired from two sources: UKB and FinnGen [20,29]. Following the process of harmonization (i.e., the standardization and integration of data from different sources into a unified dataset), a set of SNPs was assessed for each exposure variable about migraine.

The MR analyses, along with sensitivity analyses, were performed using the TwoSampleMR package in R software (version 4.2.3; R Foundation for Statistical Computing, Vienna, Austria). The analytical workflow involved retrieving genetic data for the exposures and corresponding outcome data. Subsequently, data harmonization ensured allele matching across two independent datasets, enabling the execution of MR analysis. Distinct MR analyses were carried out for migraine within the UKB and FinnGen consorts. A significance threshold of P<0.05 was applied to all MR analyses, primarily relying on the inverse variance weighted (IVW) method. We then used more conservative MR measures, including MR-Egger, which accounts for increased pleiotropy and were examined to assess potential deviations from IVW findings.

## Results

Genetic characteristics of risk factors and migraine

The number of SNP variants evaluated for each risk factor spanning from six to 93, ultimately culminating in a cumulative total of 282 SNPs across all risk factors. These genetic markers were acquired from diverse consorts, where the sample sizes exhibited considerable variability, ranging from 64,949 to 632,802 individuals per risk factor (Table 1).

**Table 1:** Synopsis of genetic risk factors: an overview SNP: single nucleotide polymorphism; GSCAN: GWAS (Genome-Wide Association Studies) & Sequencing Consortium of Alcohol and Nicotine use; UKB: UK Biobank; GIANT: Genetic Investigation of Anthropometric Traits

Exposure	Number of SNPs	Sample size	Population (consortium)
Smoking initiation	93	632,802	GSCAN
Smoking intensity	6	108,946	Within-family GWAS consortium
Maternal smoking	7	289,727	UKB
Cheese intake	65	451,486	UKB
Salad intake	22	462,933	UKB
Coffee intake	3	64,949	UKB
BMI	79	339,152	GIANT
Physical activity	7	261,055	Klimentidis et al. [10]

Characteristics of migraine variables among different populations

The migraine variable was derived from two distinct populations, namely the UKB and FinnGen. The UKB cohort consisted of 462,933 participants, among whom there were approximately 14,000 migraine cases. The FinnGen sample comprised 218,792 participants, with approximately 19,676 reporting migraine patients.

Migraine Risk in the UKB Cohort

Logistic regression showed the history of maternal smoking associated with an increased risk of migraine (OR: 1.02 for positive history versus not, 95%CI: 1.012-1.027, P = 0.004). There were statistically significant associations reflecting the reduced risk of migraine in individuals with (genetically estimated) coffee consumption (OR = 0.968 per one SD higher coffee intake, 95%CI: 0.945-0.991, P = 0.007), BMI (OR = 0.980 per single unit of BMI, 95%CI: 0.970-0.991, P <0.001), and higher physical activity (OR= 0.951, 95%CI: 0.908-0.996, P = 0.032) (Figure 1).

**Figure 2:**
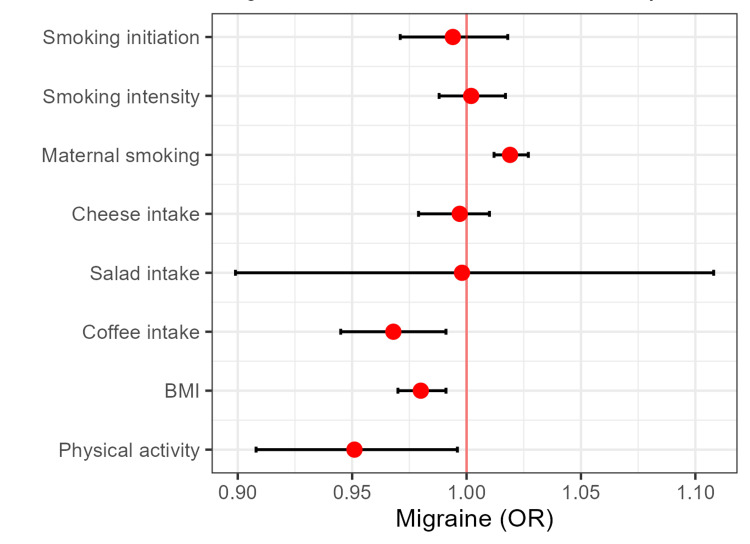
Migraine risk in the UK Biobank cohort

Migraine Risk in the FinnGen Cohort

In the FinnGen consortium, the investigation unveiled a significant relationship between genetic predisposition to cheese intake (OR = 0.765, 95%CI: 0.614-0.952, P = 0.011), salad intake (OR = 0.468, 95%CI: 0.258-0.848, 0.952, P = 0.012), and coffee consumption (OR = 0.286, 95%CI: 0.123-0.664, P = 0.004), as well as BMI (OR = 0.625, 95%CI: 0.459-0.852, P = 0.003), and decreased risk of migraine (Figure 2).

**Figure 3:**
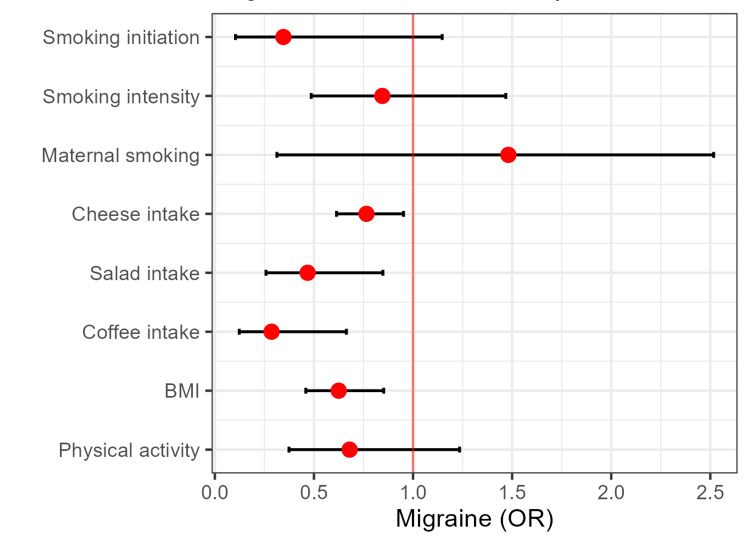
Migraine risk in FinnGen cohort

Comparison of Migraine Risk Between the UKB and FinnGen Cohorts

The comparative analysis of migraine risk between the two population cohorts reveals a consistent pattern. Notably, the risk of migraine conferred by modifiable factors in the FinnGen cohort surpasses that of the UKB cohort by nearly 47% for maternal smoking, 23% for cheese intake, 68% for coffee consumption, and 36% for BMI. This elevated magnitude of risk represents a prominent feature characterizing all the identified associations within the FinnGen dataset.

## Discussion

In this study, the causal relationship between smoking behavior and other modifiable risk factors and the risk of migraine was investigated using two population cohorts: FinnGen and UKB. We used a two-sample MR approach to assess the causal relationship between potential risk factors and the risk of migraine.

Our results revealed that increased (genetically estimated) coffee consumption cheese intake, physical activity, and lower BMI were correlated with reduced risk of migraine in both the UKB and FinnGen cohorts, which is consistent with prior studies that have shown an inverse correlation between such factors and migraine [11,30-32]. The mechanisms causing these correlations are not fully understood; they may influence the risk of migraine by affecting brain function, inflammation, or other biological pathways. There is a general recognition that fundamental lifestyle factors influence brain/physical health across the lifespan [33].

The current study results provide valuable knowledge about the causal role of smoking behavior and other modifiable risk factors in migraine development. We found that maternal smoking was linked to an increased risk of migraine in the UKB cohort (OR = 1.02, P = 0.004). The pathophysiology that causes this relationship is not fully understood, but one of the possible mechanisms is that maternal smoking may alter fetal brain development by a mechanism that leads to an increased risk of migraine later in life [34]. This finding is consistent with other research results that have shown a relationship between maternal smoking and migraine in offspring [14,34]. However, to the best of our knowledge, the current study is the first study that employed MR to establish a causal relationship between maternal smoking and migraine.

The present study corroborates the conclusions of prior studies indicating a reverse correlation among physical activity, coffee consumption, cheese intake, BMI, and migraine [11,35,36]. However, this is the first study that employed MR to establish a causal relationship between these factors and migraine.

When interpreting the current study findings, some limitations should be considered. Firstly, it was conducted in two populations of European ancestry, so the findings may not be generalizable to other populations [37]. Furthermore, not all possible confounders of the relationships between the variables of migraine risk and frequency were considered in the study. There is demonstrable and recognized selection bias in the UKB data both at baseline [38] and in terms of subsequent participation [39].

We recommend future research in independent cohorts, including those with diverse populations, to replicate our study findings in other populations. Moreover, we recommend future studies to investigate the possible pathophysiology behind the relationship between risk factors and migraine. Subsequent studies may conduct primary interventions to consider the role of dietary, adipose, and physical activity traits in lowering the risk of migraine in high-risk participants.

## Conclusions

This study implies a connection between maternal smoking and a heightened risk of migraines, whereas cheese intake, salad intake, coffee consumption, BMI, and physical activity are associated with a lower risk of migraine development. These findings might have significant implications for the advancement of effective preventive strategies and therapeutic strategies for migraine.
